# Adolescent eating disorder behaviours and cognitions: gender-specific effects of child, maternal and family risk factors

**DOI:** 10.1192/bjp.bp.114.152371

**Published:** 2015-10

**Authors:** N. Micali, B. De Stavola, G. Ploubidis, E. Simonoff, J. Treasure, A. E. Field

**Affiliations:** **N. Micali**, MD, PhD, University College London Institute of Child Health, Behavioural and Brain Sciences Unit, London; **B. De Stavola**, PhD, Centre for Statistical Methodology, London School of Hygiene and Tropical Medicine; **G. Ploubidis**, PhD, Department of Population Health, London School of Hygiene and Tropical Medicine, London; **E. Simonoff**, Department of Child and Adolescent Psychiatry, King's College London, Institute of Psychiatry and National Institute of Health Research Biomedical Research Unity for Mental Health, London; **J. Treasure**, MBBS, PhD, Eating Disorders Research Unit, Psychological Medicine, Institute of Psychiatry, King's College London, London, UK; **A. E. Field**, ScD, Division of Adolescent Medicine, Department of Medicine, Boston Children's Hospital and Harvard Medical School, Channing Division of Network Medicine, Brigham and Women's Hospital, and Department of Epidemiology, Harvard School of Public Health, Boston, Massachusetts, USA

## Abstract

**Background**

Eating disorder behaviours begin in adolescence. Few longitudinal studies have investigated childhood risk and protective factors.

**Aims**

To investigate the prevalence of eating disorder behaviours and cognitions and associated childhood psychological, physical and parental risk factors among a cohort of 14-year-old children.

**Method**

Data were collected from 6140 boys and girls aged 14 years. Gender-stratified models were used to estimate prospective associations between childhood body dissatisfaction, body mass index (BMI), self-esteem, maternal eating disorder and family economic disadvantage on adolescent eating disorder behaviours and cognitions.

**Results**

Childhood body dissatisfaction strongly predicted eating disorder cognitions in girls, but only in interaction with BMI in boys. Higher self-esteem had a protective effect, particularly in boys. Maternal eating disorder predicted body dissatisfaction and weight/shape concern in adolescent girls and dieting in boys.

**Conclusions**

Risk factors for eating disorder behaviours and cognitions vary according to gender. Prevention strategies should be gender-specific and target modifiable predictors in childhood and early adolescence.

Eating disorders have a peak of incidence at age 15–19 years,^1^ but symptoms of such disorders (behaviours and cognitions) are common in pre-adolescence and early adolescence.^1,2^ In 2012 in the UK an all-party parliamentary group on body image recommended the introduction of mandatory lessons on body image in primary and secondary schools;^3^ mandatory lessons were introduced specifically to reduce body image concerns. However, evidence that body image distortion in childhood predicts later body image and the development of full-blown eating disorder is limited to small studies,^4^ mostly of girls.^5^ Little is known about how childhood body dissatisfaction tracks into adolescent eating disorder behaviours and cognitions in boys and girls, although short-term prospective studies have suggested high stability of body dissatisfaction across ages.^6^

Prospective epidemiologic studies have highlighted thin body preoccupation, body dissatisfaction, social pressure to be thin and family history of eating disorder or eating concerns as the strongest predictors of eating disorder and disordered eating.^2,7,8^ In addition, self-esteem has been identified as a protective factor for eating disorder and eating disorder behaviours in some longitudinal studies.^2^ However, few studies of antecedents and risk factors for eating disorders and disordered eating have been conducted using samples followed up since childhood;^4^ most prospective studies in the field have enrolled adolescents and young adults. Thus relatively little is known about the role of childhood risk factors and onset of behaviours early in adolescence. Few studies have used sufficiently large population-based samples of boys and girls to examine the specificity of risk factors across genders. This lack of knowledge affects the development of effective public health interventions and prevention, given that these require not only evidence of modifiable causal factors and/or early manifestations of disorders but also knowledge of when to intervene and whom to target. Understanding the latter two is extremely important for clinical and public health purposes.

The purpose of this study was first to investigate the prevalence of eating disorder behaviours (dieting, bingeing and purging) and cognitions (body dissatisfaction, weight and shape concern and pressure to lose weight) at age 14 years in a UK population-based sample of boys and girls and to understand them in the context of prevalence studies across the world, and second to determine whether previously identified childhood individual risk factors (body dissatisfaction, weight and self-esteem) and parental risk factors (maternal history of an eating disorder, and socioeconomic disadvantage in early and late childhood), using a biopsychosocial risk model, predict eating disorder behaviours and cognitions at age 14 years. Given gender differences in the relationship between weight and body dissatisfaction,^9–11^ which show that the relationship between body dissatisfaction and body mass index (BMI) differs between genders, we were particularly interested in understanding whether weight and body dissatisfaction in childhood acted synergistically (in interaction) or independently in their prospective association with adolescent eating disorder behaviours and cognitions and whether it differed in boys and girls.

## Method

The Avon Longitudinal Study of Parents and Children (ALSPAC) is a population-based prospective study of women and their children.^12^ All pregnant women living in the geographical area of Avon, UK, who were expected to deliver their baby between 1 April 1991 and 31 December 1992, were invited to take part in the study. The children from 14 541 pregnancies were enrolled; 13 988 children were alive at 1 year. An additional 713 children were enrolled later on in childhood (phases 2 and 3).^13^ All women gave informed and written consent. At age 14 years 10 137 children enrolled in the study at birth and 444 enrolled as part of phases 2 and 3 were eligible for follow-up,^13^ and were sent questionnaires. Questionnaires were returned by 6281 adolescents (59.4% of those eligible). Among the 72 twin pairs, one twin per pair was randomly excluded from the available sample. Completed questionnaires were available for 6140 adolescents (5782 originally enrolled and 268 enrolled at age 7 years). Missing items slightly reduced the sample available for each outcome. The study website contains details of all the available data through a fully searchable data dictionary (http://www.bris.ac.uk/alspac/researchers/data-access/data-dictionary/). Ethical approval for the study was obtained from the ALSPAC ethics and law committee and the local research ethics committees.

### Eating disorder behaviours

Data on eating disorder behaviours at age 14 years were collected using questions adapted from the Youth Risk Behavior Surveillance System questionnaire,^14^ enquiring about the previous year. Binge eating was assessed using a two-part question. Participants were first asked about the frequency during the past year of eating a very large amount of food; those who answered ‘yes’ were directed to a follow-up question that asked whether they felt out of control during these episodes, as if they could not stop eating even if they wanted to. Binge eating was coded if adolescents answered ‘yes’ to both questions. Purging was assessed by asking how often in the past year participants made themselves sick or used laxatives to lose weight or avoid gaining weight. Both questions have been validated in an adolescent population-based sample.^15^ Dieting was assessed with one question: ‘During the past year, did you go on a diet to lose weight or keep from gaining weight?’

### Eating disorder cognitions

Weight and shape concern at age 14 years was assessed using three questions from the McKnight Risk Factor Survey:^16^ ‘In the past year, how happy have you been with the way your body looks?’; ‘In the past year, how much has your weight made a difference to how you feel about yourself?’; ‘In the past year, how much have you worried about gaining a little weight (as little as 1 kilo)?’. Four responses on a Likert scale were used (from ‘very unhappy’ or ‘not at all’ to ‘very happy’ or ‘a lot/all the time’). The three questions were summed to generate a continuous score. Cronbach's alpha in this sample was 0.66. Pressure to lose weight was assessed using an adapted version of the Perceived Sociocultural Pressure Scale.^17^ Participants were asked to rate the statements: ‘I have felt pressure to lose weight: (a) from my friends, (b) from my family, (c) from the boys/girls I have gone out with, and (d) from the media (e.g. TV, magazines)’ on a four-point Likert scale (from ‘not at all’ to ‘a lot’). Items were summed to obtain a total score; Cronbach's α = 0.71 in this sample. Body dissatisfaction was assessed using the Body Dissatisfaction Scale;^18^ this scale asks individuals to rate their satisfaction with nine body parts on a Likert scale, from ‘extremely satisfied’ to ‘extremely dissatisfied’. Questions were slightly adapted in the female questionnaire version (‘body build’ in the male version was replaced by ‘breasts’) following feedback from the ALSPAC teenage advisory panel. A continuous score was derived, with higher values indicating higher dissatisfaction. Cronbach's á was 0.92 for the male version and 0.91 for the female version in this study. Missing items on each of the three eating disorder cognitions scales were prorated for individuals who had fewer than 20% items missing in each scale.

### Childhood factors

#### Body dissatisfaction

Body dissatisfaction at age 10.5 years was assessed using gender-appropriate Stunkard figure rating scales.^19^ Children were asked to rate their current and desired body image. A body dissatisfaction score was generated by subtracting the ideal body shape chosen from the current perceived body image (higher scores indicate higher body dissatisfaction). Data were available for 5097 adolescents who participated in the survey at age 14 years.

#### Body mass index

Body mass index (BMI) at age 10.5 years was obtained from supplementing data on weight and height collected from face-to-face assessments (available for 4749 children providing data at age 14 years) with maternally reported weight and height obtained from questionnaires at the same time point (available for 3241 children); the correlation between maternally reported and objective BMI in those with both measurements was 0.91. Age- and gender-adjusted BMI *z*-scores (using UK references) were obtained from the Stata user-defined program Z-anthro.^20,21^ The BMI was available for 5291 participants.

#### Self-esteem

Children completed a shortened form of Harter's Self-perception Profile for Children as part of a face-to-face assessment at age 8.5 years.^22^ This instrument consisted of the 12 items from the global self-worth and scholastic competence subscales of the measure. For the purpose of this study we only used the global self-worth subscale (higher scores indicate higher self-esteem). Data were available for 4484 participants who provided adolescent data.

### Family factors

#### Socioeconomic disadvantage

Data on financial problems were obtained from maternal reports at regular intervals throughout childhood by means of questionnaires. We derived a three-level variable indicating no financial problem, early childhood financial problems (child age 0–5 years) and late childhood financial problems (age 5–10 years); this variable has previously been shown to predict mental health outcomes in this cohort.^23^ Data were available for 5703 of the 6140 study adolescents.

#### Maternal eating disorder

All mothers taking part in the ALSPAC study were asked during pregnancy whether they had ever experienced anorexia nervosa or bulimia nervosa,^24^ and the question was repeated when the index child was 7 years old. Given the difficulty in ascertaining timing of occurrence of maternal disorder, women were classified as having ever (up to child age 7 years) experienced anorexia nervosa, bulimia nervosa or both conditions.^24^ Data were available for 5102 mothers of participating adolescents.

### Confounders

Data on maternal age at enrolment in the cohort and parental social class were obtained by questionnaire at enrolment. Adolescent BMI *z*-scores at age 14 years were obtained as above from a combination of objective BMI collected at a face-to-face assessment at mean age 13.7 years (available for 4602 adolescents) and self-reported BMI at 14 years (available for 3305 adolescents); the correlation of self-reported and objective BMI was 0.89.

### Statistical analysis

Descriptive data are presented as means and standard deviations for continuous outcomes and as frequencies and percentages for categorical outcomes. The prevalence of eating disorder behaviours and cognitions was compared across genders using chi-squared tests (for categorical outcomes) and analysis of variance (for continuous outcomes). All continuous outcomes were investigated for normality. Univariable logistic and linear regression models were used to investigate the association between each predictor and outcome stratified by gender. Predictors associated with outcomes at the *P*<0.1 level were subsequently included in multivariable models. Multivariable models were adjusted for *a priori* confounders (maternal age at enrolment, parental lowest combined social class and adolescent BMI at 14 years). We adjusted all analyses for BMI at age 14 years to control for confounding by this factor, which is positively associated with later weight status and with eating disorder behaviours and cognitions, the outcomes of our study. All regression models included an interaction between childhood body dissatisfaction and childhood BMI (treated as continuous), given that this was a specific aim. Collinearity between predictors in multivariable analyses was assessed by calculating the variance inflation factor.^25^ The numbers of adolescents available for analyses varied slightly owing to missing data on individual questions for the age 14 questionnaire. Because of missing data on covariates, multiple imputation by chained equation,^26^ with ten imputation sets, was implemented in Stata version 12 assuming data missing at random.^27^ The imputation included all variables included in the analyses and predictors of missingness (maternal age at enrolment, parental lowest combined social class and child BMI at 14 years). Results obtained after imputation were similar to those found when analysing the complete records only; therefore results obtained from multiple imputation are reported.

## Results

Table 1 shows the distribution of sociodemographic and predictor variables, stratified by gender.

**Table 1:** Distribution of sociodemographic and predictor variables

	Girls	Boys
*Sociodemographic variables*		
Age at assessment, months		
*n*	3404	2736
Mean (s.d.)	168.34 (2.14)	168.67 (2.45)
BMI at 14 years, kg/m^2^		
*n*	2799	2348
Mean (s.d.)	20.68 (3.47)	19.90 (3.27)
Parental lowest combined social class at enrolment		
*n*	2703	2236
Manual, *n* (%)	435 (16.09)	302 (13.51)
Non-manual, *n* (%)	2268 (83.91)	1934 (86.49)
Maternal age at enrolment, years		
*n*	3240	2632
Mean (s.d.)	28.95 (4.57)	29.41 (4.60)

*Predictors*		
Child factors		
Self-esteem at age 8½ years, *n*	2454	2030
Global self-worth score: mean (s.d.)	19.45 (3.28)	19.26 (3.43)
Body dissatisfaction at age 10 years, *n*	2829	2268
Mean (s.d.)	0.26 (0.69)	0.08 (0.63)
BMI *z*-score at age 10 years, *n*	2884	2407
Mean (s.d.)	0.23 (1.16)	0.36 (1.17)
Maternal eating disorder, lifetime, *n*	2768	2334
No eating disorder, *n* (%)	2610 (94.29)	2205 (94.47)
Anorexia nervosa, *n* (%)	45 (1.63)	49 (2.10)
Bulimia nervosa, *n* (%)	88 (3.18)	58 (2.49)
Anorexia and bulimia, *n* (%)	25 (0.90)	22 (0.94)
Family economic disadvantage, *n*	3119	2584
Financial problems child age 0–5 years, *n* (%)	619 (19.85)	476 (18.42)
Financial problems child age 6–10 years, *n* (%)	337 (10.80)	296 (11.46)

BMI, body mass index.

### Prevalence of eating disorder behaviours and cognitions

Table 2 summarises the distribution of the outcomes across genders: body dissatisfaction, weight and shape concern and reported pressure to lose weight. These were all significantly higher in girls compared with boys. When dichotomised in line with Field *et al*,^28^ 4.7% of boys and 11.4% of girls reported high weight and shape concern. Nearly a fifth of girls (18%) *v.* 3% of boys reported feeling ‘quite a lot/a lot’ of pressure from the media to lose weight. Almost 40% of girls and 12% of boys reported dieting in the year prior to assessment; 7.5% of girls and 3.5% of boys engaged in bingeing, but few adolescents reported engaging in purging behaviours (2.4% of girls and 0.8% of boys). Frequent dieting (always or often in the past year) was reported by 7.6% of girls and 1.6% of boys.

**Table 2:** Prevalence of eating disorder cognitions and behaviours at age 14 years

Outcomes	Girls	Boys		
Body dissatisfaction				
Available *n*	3372	2669		
Mean (s.d.)	23.7 (8.0)	19.7 (7.0)	*F*(1,6039) = 433.15	*P*< 0.0001

Weight and shape concern				
Available *n*	3375	2665		
Mean (s.d.)	5.88 (1.99)	4.70 (1.46)	*F*(1,6038) = 648.95	*P*< 0.0001

Perceived pressure to lose weight				
Available *n*	3324	2652		
Mean (s.d.)	4.76 (1.67)	4.24 (0.95)	*F*(1,5974) = 379.87	*P*< 0.0001

Pressure to lose weight from the media				
Available *n*	3365	2682		
Not at all, *n* (%)	1833 (54.47)	2350 (87.62)	χ^2^ = 851.49	*P*< 0.0001
A little, *n* (%)	935 (27.79)	252 (9.40)		
Quite a lot/a lot, *n* (%)	597 (17.74)	80 (2.98)		

Dieting in the past year				
Available *n*	3374	2688	χ^2^ = 574.43	*P*< 0.0001
*n* (%)	1321 (39.15)	333 (12.40)		

Bingeing in the past year				
Available *n*	3365	2687	χ^2^ = 44.26	*P*< 0.0001
*n* (%)	254 (7.55)	95 (3.54)		

Purging in the past year				
Available *n*	3349	2657	χ^2^ = 23.52	*P*< 0.0001
*n* (%)	81 (2.42)	21 (0.79)		

#### Co-occurrence of eating disorder behaviours

**Fig. 1:**
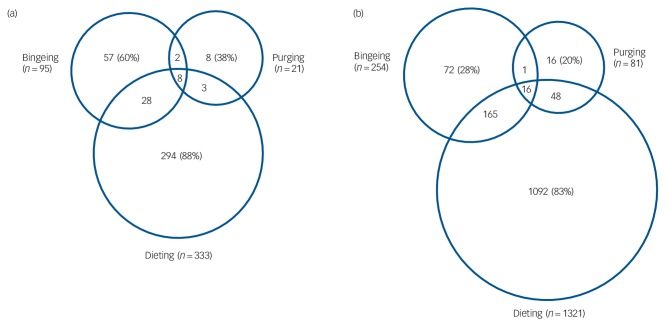
Co-occurrence of eating disorder behaviours across genders: (a) boys (*n* = 2688); (b) girls (*n* = 3374).

The majority (>80%) of boys and girls who dieted did not engage in bulimic behaviours (Fig. 1). Although few adolescents reported purging, 38% of boys who purged did not engage in the other two behaviours (dieting and/or bingeing); and 20% of girls who purged did not diet or binge. A very low proportion of boys and girls engaged in both bingeing and purging: 0.4% of boys and 0.5% of girls. Bingeing was differentially comorbid with dieting and purging in girls and boys. Although 60% of boys who binged did not engage in other eating disorder behaviours, this percentage was much lower in girls – only 28% of girls who binged did not engage in other eating disorder behaviours (Fig. 1).

### Predictors of eating disorder cognitions

#### Adolescent body dissatisfaction

In univariable models higher adolescent body dissatisfaction was strongly predicted by childhood body dissatisfaction and higher BMI at age 10 years (online Table DS1). Higher self-esteem in childhood predicted lower adolescent body dissatisfaction in boys and girls. Among maternal lifetime eating disorder categories only a history of both anorexia and bulimia nervosa significantly predicted greater adolescent body dissatisfaction in girls but not in boys. Family economic disadvantage in early and late childhood was associated with body dissatisfaction at age 14 years across genders (online Table DS1). Multivariable analyses in girls showed childhood body dissatisfaction and its interaction with childhood BMI predicted higher adolescent body dissatisfaction (for body dissatisfaction, B = 1.42, 95% CI 0.75–2.10, *P*≤0.0001; for body dissatisfaction × BMI, B = 0.90, 95% CI 0.50–1.30, *P*≤0.0001; Table 3). Higher self-esteem was prospectively associated with lower body dissatisfaction scores at age 14 years (B=−0.14, 95% CI −0.24 to −0.03, *P*≤0.001). Maternal lifetime anorexia and bulimia nervosa predicted higher body dissatisfaction (B = 5.63, 95% CI 1.83–9.42, *P*≤0.001). Multivariable analyses in boys showed a slightly different pattern: childhood body dissatisfaction was a significant predictor of adolescent body dissatisfaction only in interaction with BMI at age 10 years (B = 1.76, 95% CI 1.40–2.13, *P*≤0.0001). As in girls, higher self-esteem predicted lower adolescent body dissatisfaction scores (Table 3).

**Table 3:** Child, maternal and social predictors of body dissatisfaction, weight and shape concern and pressure to lose weight at 14 years: linear regression coefficients and 95% CIs from multivariable models^a^

	Body dissatisfaction		Weight and shape concern		Pressure to lose weight	
	Girls (*n* = 1786)	Boys (*n* = 1552)	Girls (*n* = 1786)	Boys (*n* = 1551)	Girls (*n* = 2137)	Boys (*n* = 1543)
Body dissatisfaction (age 10 years)	1.42*** (0.75, 2.10)	−0.23 (−0.90, 0.43)	0.29*** (0.13,0.46)	0.04 (−0.10, 0.18)	0.24** (0.10, 0.39)	0.11 (−0.20, 0.24)

BMI z-scores (age 10 years)	0.28 (−0.31, 0.87)	−0.002 (−0.59, 0.58)	0.05 (−0.09,0.20)	0.07 (−0.04, 0.20)	0.20** (0.07, 0.34)	0.05 (−0.05, 0.17)

Body dissatisfaction × BMI z-scores (age 10 years)	0.90*** (0.50, 1.30)	1.76*** (1.40, 2.13)	0.21*** (0.11,0.31)	0.26*** (0.18, 0.33)	0.08 (−0.01, 0.17)	0.22* (0.15, 0.29)

Self-esteem	−0.14** (−0.24, −0.03)	−0.16** (−0.25, −0.01)	−0.01(−0.03,0.02)	−0.02* (−0.04, 0.001)		−0.20* (−0.03, 0.001)

Maternal lifetime eating disorder						
Anorexia nervosa	−0.34 (−3.70, 3.00)		0.13 (−0.69, 0.95)	0.33(−0.17, 0.83)		
Bulimia nervosa	1.49 (−0.93, 3.91)		−0.10 (−0.70, 0.49)	0.11(−0.35, 0.57)		
Anorexia and bulimia	5.63** (1.83, 9.42)		1.96*** (1.03, 2.90)	0.54(−0.18, 1.26)		

Financial problems:						
Early	0.46 (−0.40, 1.33)	0.78 (−0.10, 1.63)	0.10 (−0.11, 0.31)	0.06 (−0.12, 0.24)	0.10 (−0.09, 0.30)	0.05 (−0.11, 0.21)
Late	−0.38 (−1.51, 0.74)	0.44 (−0.57, 0.87)	0.03 (−0.24, 0.31)	0.16 (−0.06, 0.38)	0.28* (0.02, 0.54)	0.09 (−0.10, 0.29)

a. Adjusted for maternal age at enrollment, parental social class and adolescent body mass index (BMI) at age 14, obtained from multiple imputation.

* P ≤ 0.05

** P ≤ 0.001

*** P ≤ 0.0001.

*Post hoc* analyses showed that among girls with high body dissatisfaction at 14 years (top decile), 55% had high body dissatisfaction (top decile) at 10 years of age; in boys, 45% of those with high body dissatisfaction at 14 years (top decile) had high body dissatisfaction (top decile) at 10 years of age.

#### Weight and shape concern

Weight and shape concern was significantly associated with most predictors across genders in univariable analyses (online Table DS1). Multivariable analyses revealed differential effects by gender: childhood body dissatisfaction and maternal lifetime anorexia and bulimia were prospectively associated with higher weight and shape concern at age 14 years in girls (respectively B = 0.29, 95% CI 0.13–0.46; B = 1.96, 95% CI 1.03–2.90; *P*≤0.0001) but not in boys (Table 3). Although the interaction between body dissatisfaction and BMI at age 10 years predicted higher weight and shape concern across genders (in girls B = 0.21, 95% CI 0.11–0.31; in boys B = 0.26, 95% CI 0.18–0.33), higher self-esteem remained predictive of lower weight and shape concern scores only in boys (Table 3).

#### Perceived pressure to lose weight

Perceived pressure to lose weight was associated with childhood body dissatisfaction, BMI and late childhood family economic disadvantage (Table 3; online Table DS1). Multivariable analyses showed a different pattern of association across genders. Among girls childhood body dissatisfaction and higher BMI *z*-scores were prospectively associated with the outcome, whereas in boys only the interaction between these two variables was predictive of higher perceived pressure to lose weight. For girls late childhood family economic disadvantage retained its effect in multivariable models. Among boys higher self-esteem was prospectively associated with lower perceived pressure to lose weight (Table 3).

### Eating disorder behaviours

#### Dieting

In univariable analyses dieting was predicted by childhood body dissatisfaction and childhood BMI *z*-scores, but not self-esteem (online Table DS2). Maternal lifetime anorexia and bulimia predicted dieting in boys but not in girls. Similarly, family economic disadvantage was prospectively associated with dieting in boys. In multivariable analyses, among girls childhood body dissatisfaction and BMI *z*-scores remained prospectively associated with dieting at age 14 years (respectively OR=1.36, 95% CI 1.12–1.66; OR=1.36, 95% CI 1.16–1.60) with no evidence of interaction (Table 4). Among boys, a one-point increase in BMI at age 10 years corresponded to a 80% increased odds of dieting at 14 years (OR=1.80, 95% CI 1.33–2.44); boys whose mothers had lifetime anorexia and bulimia were approximately five times more likely to diet at age 14 years (OR=4.98, 95% CI 1.54–16.1).

**Table 4:** Child, maternal and social predictors of dieting, bingeing and purging at 14 years: odds ratios and 95% CIs from multivariable models^a^

	Dieting		Bingeing		Purging	
	Girls (*n* = 2182)	Boys (*n* = 1703)	Girls (*n* = 1934)	Boys (*n* = 1842)	Girls (*n* = 2212)	Boys (*n* = 1576)
Body dissatisfaction (age 10 years)	1.36* (1.12, 1.66)	1.16 (0.74, 1.81)	1.31 (0.90, 1.91)		0.80 (0.48, 1.33)	3.3*** (0.80, 13.00)

BMI z-scores (age 10 years)	1.36** (1.16, 1.60)	1.80** (1.33, 2.44)	0.98 (0.70, 1.38)	0.83 (0.53, 1.31)	0.81 (0.50, 1.30)	0.85 (0.23, 3.17)

Body dissatisfaction × BMI z-scores (age 10 years)	0.97 (0.85, 1.10)	1.10 (0.88, 1.38)	0.95 (0.76, 1.20)	1.32* (1.10, 1.61)	1.28** (1.02, 1.61)	0.94 (0.43, 2.04)

Self-esteem (age 8 years)			0.96 (0.91, 1.01)			0.81** (0.68, 0.96)

Maternal lifetime eating disorder						
Anorexia nervosa		0.41 (0.09, 1.81)				
Bulimia nervosa		0.88 (0.31, 2.49)				
Anorexia and bulimia		4.98* (1.54, 16.1)				

Financial problems						
Early		1.10 (0.71, 1.70)	1.48 (0.95, 2.28)	2.25* (1.22, 4.14)		
Late		1.42 (0.88, 2.28)	1.06 (0.57, 1.97)	1.95 (0.93, 4.10)		

a. Adjusted for maternal age at enrollment, parental social class and adolescent body mass index (BMI) at age 14, from multiple imputation.

* *P* ≤ 0.001

** *P* ≤ 0.0001

*** *P* ≤ 0.1.

#### Bingeing

Among girls childhood body dissatisfaction and childhood BMI were prospectively associated with bingeing at age 14 years, and higher self-esteem was associated with lower odds of bingeing (Table 4). Among boys, childhood BMI *z*-scores were prospectively associated with bingeing at age 14 years. Family economic disadvantage was associated with bingeing across genders. In the multivariable analysis, none of the predictors was significant among girls. However, among boys, childhood body dissatisfaction in interaction with childhood BMI (OR = 1.32, 95% CI 1.10–1.61) and early family economic disadvantage (OR = 2.25, 95% CI 1.22–4.14) were prospectively associated with bingeing at age 14 years (Table 4).

#### Purging

Childhood body dissatisfaction and BMI were prospectively associated with purging across genders in univariable analyses (online Table DS2). Higher self-esteem at age 8 years was associated with lower odds of purging in boys. Although boys and girls with maternal lifetime eating disorder had high odds of purging (maternal lifetime anorexia for boys and girls and lifetime bulimia for boys), these results did not reach statistical significance, probably owing to low prevalence of predictors and outcome. Multivariable analyses showed an effect of childhood body dissatisfaction in interaction with childhood BMI in predicting purging among girls (OR = 1.28, 95% CI 1.02–1.61) and a tripling of odds associated with childhood body dissatisfaction (OR = 3.3, 95% CI 0.83–13.03; *P* = 0.09) among boys. Higher self-esteem was prospectively associated with lower odds of purging at age 14 years among boys (Table 4).

## Discussion

Among 6140 adolescents aged 14 years from the ALSPAC cohort, dieting and bingeing in the year prior to assessment were common among girls (39% had dieted and 8% binged) but less common among boys (12% had dieted and 3.5% had binged). Purging was uncommon among both girls (2.4%) and boys (0.8%). As expected, gender differences were also observed for level of eating disorder cognitions. Childhood psychological, physical and parental or family factors were observed to predict eating disorder behaviours and cognitions 4–6 years later. Associations varied by gender.

### Main findings

This is the largest study to date of eating disorder cognitions and behaviours in the UK. As expected, eating disorder cognitions among young UK teenagers were higher in girls than in boys. Levels of body dissatisfaction in our sample were comparable with those reported in US adolescents.^11,29^ Mean weight and shape concern was higher in girls than in boys; the prevalence of high weight and shape concern was slightly higher than, but broadly comparable with, a population-based US study that used the same measure.^28^ Perceived pressure to lose weight from the media was reported by about a fifth of girls, with notable gender differences. In relation to eating disorder behaviours, levels reported in our study are comparable with those reported among adolescent girls in an inner-city sample in the UK.^30^ About 8% of girls were frequent dieters in our sample, consistent with US prevalence figures.^31^ Bingeing and purging were less prevalent in our study than in a recent survey of secondary schools in Australia,^32^ and a study on adolescents and young adults in Norway;^33^ however, those studies included older adolescents and used different assessments, which might explain the differences.

This is the largest prospective investigation of the association of childhood physical, psychological and family predictors of eating disorder behaviours and cognitions 4–6 years later among young adolescent boys and girls. We identified a strong effect of childhood body dissatisfaction on adolescent body dissatisfaction, weight and shape concern and pressure to lose weight and dieting in girls, as previously reported.^18,34^ In contrast, in boys the effect of body dissatisfaction on later eating disorder outcomes was seen mainly in interaction with BMI. Boys with high BMI and high childhood body dissatisfaction had higher levels of eating disorder cognitions and behaviours, but there was no association with childhood body dissatisfaction among leaner boys. All analyses controlled for BMI concurrent with eating disorder behaviours and cognitions, thus the results suggest that regardless of weight status at age 14 years childhood BMI was predictive of eating disorder cognitions and behaviours in adolescence. Previous studies have shown a gender difference in the association between body dissatisfaction and BMI,^10^ but none to our knowledge has demonstrated a gender-specific role of childhood body dissatisfaction in predicting later disordered eating. This differential effect might be due to several reasons, including gender-specific patterns of body dissatisfaction,^35^ which are strongly linked to cultural and social expectations. It is beyond the scope of this paper to discuss this at length; however, it is possible that given fewer social pressures on males to be thin, body dissatisfaction at an early age has a ‘negative’ effect only on boys who are overweight or obese, whereas even lean girls may experience pressure to be thin. This gender difference is particularly important for prevention strategies, given that body dissatisfaction might need to be targeted differently across genders.

The persistence and negative later effects of childhood body dissatisfaction point to a need for early prevention, at primary school age. To our knowledge no previous study has investigated whether pressure to lose weight is predicted by late childhood body dissatisfaction or weight status. Our findings show that childhood body dissatisfaction and weight have in fact a predictive role in early adolescent report of pressure to lose weight. This suggests that contrary to previous reports, some children (vulnerable because of early body dissatisfaction and higher weight) might be more vulnerable to feeling under pressure from media, family and peers. Similarly, the role of high self-esteem in being prospectively associated with lower levels of eating disorder behaviours and cognitions confirms previous findings from population-based mixed gender samples.^36,37^ Interestingly, in our study the protective effect of self-esteem for eating disorder behaviours and cognitions was stronger among boys.

Maternal history of anorexia and/or bulimia nervosa was predictive of high levels of body dissatisfaction and weight and shape concern in girls and dieting in boys. The effect was more pronounced for children of women who reported both anorexia and bulimia over their lifetime (up to child age 7 years). Despite this group being small, women in this group had more severe symptoms compared with those who reported only one lifetime disorder in our previous investigations of this sample.^24^ There is increasing evidence on the intergenerational effects of maternal eating disorder on child psychopathology,^38^ childhood eating,^39^ and adolescent eating disorder.^2^ We showed a gender-specific effect, but small sample size might have resulted in low power to detect true differences and led to false negatives. Surprisingly, the role of family adversity (in this case economic disadvantage) in relation to later eating disorder cognitions and behaviours was minimal in multivariable analyses. Previous studies have shown a small effect of family adversity in relation to later eating disorder.^34^

### Strengths and limitations

Our findings need to be interpreted taking into account relevant strengths and limitations. This is the first study in the UK to investigate the prevalence of eating disorder behaviours and cognitions and childhood risk and protective factors in a population-based sample broadly representative of the overall UK population. The large sample size and the possibility of stratifying our sample by gender allowed a clear identification of gender-specific patterns that have been insufficiently studied in prior research. In contrast to many previous studies that investigated risk factors at later ages (when prodromal eating disorder symptoms are likely to confound results) we were able to study very early risk factors and psychological predictors. However, despite our large sample size we were able to estimate the effect of uncommon predictors on uncommon outcomes with less precision, potentially resulting in some false negatives and near-significant results. As is common in longitudinal birth cohorts, this study suffered from some attrition, with selective loss of more disadvantaged families,^13^ which might affect generalisability and may explain why childhood adversity was not a risk factor in our analyses. However, the adoption of multiple imputation methods to include in the analyses children with incomplete records under the missing at random assumption may have removed some of the potential selection bias. We did not have data on eating disorder behaviours or cognitions in late childhood so we could not adjust for these, but it is likely that their prevalence would have been very low in late childhood.

### Clinical implications

This study suggests that eating disorder cognitions and behaviours are common in young adolescents, especially girls. The identified high prevalence of some eating disorder behaviours in boys highlights a need for awareness. This has important clinical implications, as many of these adolescents might progress to a full-blown eating disorder. Childhood body dissatisfaction was a strong predictor of eating disorder cognitions and behaviours 4 years later, differentially in boys and girls. Similarly, high childhood self-esteem was protective for disordered eating, especially in boys. Future research should investigate further gender-specific risk pathways. Later waves of data collection on eating disorder behaviours and cognitions in this sample will allow determination of differential effects of risk factors at various ages. Preventive efforts need to take into account the differential effect of psychological risk factors across genders, and will need to target young children as well as older adolescents. In view of our findings, targeted prevention strategies might be preferable to universal ones, given the differences in risk across weight status and gender – for example by targeting boys who are overweight or obese and have body dissatisfaction and girls with high body dissatisfaction in late childhood.
